# Supplementary material for: The Evaluation of Videos about Branched-Chain Amino Acids Supplements on YouTube TM: A Multi-Approach Study
Source: Int J Environ Res Public Health. 2022 Dec 11;19(24):16659. doi: 10.3390/ijerph192416659 (PMC9779575; doi:10.3390/ijerph192416659)
Supplement: Supplementary file 1 [file ijerph-19-16659-s001.zip › ijerph-2031235-supplementary.pdf]

Supplementary Materials

Table S1. The questions of DISCERN instrument.

	No		Partially		Yes
	1	2	3	4	5
1. Are the aims clear?					
2. Does it achieve its aims?					
3. Is it relevant?					
4. Is it clear what sources of information were used to compile the publication (other than the author or producer)?					
5. Is it clear when the information used or reported in the publication was produced?					
6. Is it balanced and unbiased?					
7. Does it provide details of additional sources of support and information?					
8. Does it refer to areas of uncertainty?					
9. Does it describe how each treatment works?					
10.Does it describe the benefits of each treatment?					
11. Does it describe the risks of each treatment?					
12. Does it describe what would happen if no treatment is used?					
13. Does it describe how the treatment choices affect overall quality of life?					
14. Is it clear that there may be more than one possible treatment choice?					
15. Does it provide support for shared decision-making?					
	Low		Moderate		High
	Serious or extensive shortcomings		*Potentially important but not serious shortcomings*		*Minimal shortcomings*
	1	2	3	4	5
16. Based on the answers to all of the above questions, rate the overall quality of the publication as a source of information about treatment choices					

http://www.discern.org.uk/discern_instrument.php (accessed on 15 October 2022).

Table S2. Journal of the American Medical Association (JAMA) benchmark criteria [35].

Criteria	Description	No	Yes
**Authorship**	Authors, contributors, affiliations, and credentials should be listed	0	1
**Attribution**	References, sources, and relevant copyright information should be listed	0	1
**Disclosure**	Web site ownership, sponsorship, advertising, commercial funding, underwriting and potential conflict of interests should be listed	0	1
**Currency**	Date of original posting and dates of updates should be indicated	0	1

Table S3. Global Quality Score criteria [53].

**Description**	**Global Quality Score**
Poor quality, poor flow, most information missing, not useful for viewers	1
Generally poor quality and poor flow, some information listed but many important topics missing, of very restricted use to viewers	2
Moderate quality, suboptimal flow, some important information is adequately discussed, somewhat useful for viewers	3
Good quality and generally good flow, most relevant information is covered, but some topics not listed, useful for viewers	4
Excellent quality, excellent flow, very useful for viewers	5
